# Children’s Self-Regulation in Cultural Contexts: The Role of Parental Socialization Theories, Goals, and Practices

**DOI:** 10.3389/fpsyg.2017.00923

**Published:** 2017-06-06

**Authors:** Jorge M. Jaramillo, María I. Rendón, Lorena Muñoz, Mirjam Weis, Gisela Trommsdorff

**Affiliations:** ^1^Facultad de Psicología, Universidad Santo TomásBogotá, Colombia; ^2^Departamento de Psicología, Universidad de ChileSantiago, Chile; ^3^Centre for International Student Assessment (ZIB), TUM School of Education, Technical University of MunichMunich, Germany; ^4^Developmental and Cross-Cultural Psychology, University of KonstanzKonstanz, Germany

**Keywords:** self-regulation, socialization theories, socialization goals and practices, parenting, child development, cultural contexts

## Abstract

Self-regulation is a complex multidimensional construct which has been approached mainly in Western cultural contexts. The present contribution examines the importance of considering the culture-sensitive nature of self-regulation by reviewing theory and research on the development of children’s self-regulation in different cultural contexts. This review of theory and research allows to suggest that widely shared values in a cultural group influence parental socialization theories, goals, and practices, which in turn have an impact on how children learn to self-regulate, the forms of self-regulation they develop, and the goals associated with self-regulation. Thus, this article concludes that more specific research is required to relate both the developmental and the cultural aspects of children’s self-regulation.

## Introduction

Self-regulation is a complex construct that has raised the interest of lifespan researchers as it has been shown to play a role in many dimensions of daily life (McClelland et al., 2010; Zhou et al., 2012). Its importance for children’s socioemotional and cognitive development has been highlighted by many researchers (McClelland and Tominey, 2011; Nozadi et al., 2015; Montroy et al., 2016; Weis et al., 2016). For instance, it has been documented that optimally self-regulated children tend to be socially competent (Eisenberg et al., 2016), whereas children with poor self-regulatory skills are at risk of experiencing peer rejection and academic difficulties (McClelland and Tominey, 2011). Self-regulation is also important for school readiness as starting school constitutes a critical developmental period in which children are involved in more structured and academically oriented environments (Cadima et al., 2015; Montroy et al., 2016). In fact, self-regulation has been linked to the development of language, mathematics, reading, and literacy skills (e.g., von Salisch et al., 2015; Bohlman and Downer, 2016; Lin et al., 2016). Moreover, from a lifespan perspective, self-regulation has lifelong consequences as it has been shown to predict life satisfaction, social behavior, physical health, and overall quality of life (Moffit et al., 2013). Although, self-regulation has been approached mainly in Western contexts, processes of self-regulation might differ across cultural contexts. According to Trommsdorff’s (2009) “Cultural Model of Agency and Self-Regulation”, the development of self-regulation might vary according to the cultural context in which the individual is embedded.

The purpose of this paper is to examine the role of cultural contexts for the development of self-regulation by reviewing studies on the relations between parental socialization theories, goals, and practices and children’s self-regulation in diverse cultural settings.

## The Conceptualization of Self-Regulation

Self-regulation is defined as “the self’s capacity for altering its behaviors……[which] greatly increases the flexibility and adaptability of human behavior, enabling people to adjust their actions to a remarkably broad range of social and situational demands” (Baumeister and Vohs, 2007, p. 115). Thus, self-regulation includes cognitive, behavioral, temperamental, and socioemotional components as it involves focusing and maintaining attention, initiating or inhibiting actions, thoughts, and emotions as well as monitoring the results, to achieve a particular goal (McClelland et al., 2010, 2015; McClelland and Cameron, 2011; Blair et al., 2015; Kelley et al., 2015).

The cognitive dimension of self-regulation has been discussed from the perspective of executive functions, which encompass the ability to hold information in the working memory, to inhibit dominant or automatic responses, and to flexibly shift the focus of attention (Blair, 2016; Montroy et al., 2016). Hence, executive functions imply the voluntary regulation of cognitions and behaviors in a purposeful and non-reactive way (Nozadi et al., 2015). Herewith, executive functions constitute an important cognitive skill, allowing to make adaptive changes in the physical and social environment (see Moriguchi et al., 2012).

Researchers have recognized that temperamental and socioemotional aspects of self-regulation are not separated from cognitive abilities (Blair and Raver, 2015). Self-regulation also includes automatic bottom-up processes such as the modulation of emotional reactivity through attentional mechanisms (Blair and Raver, 2015; McClelland et al., 2015). In fact, emotion regulation has been defined as automatic or effortful processes that serve the function of modulating emotional experiences and expressions as well as monitoring, evaluating, and modifying their intensity and duration to accomplish goals in a particular context, for which the individual should apply social rules and standards of behavior (Thompson, 1994; Campos et al., 2004; Eisenberg and Spinrad, 2004; Thompson et al., 2008; Calkins and Leerkes, 2011; Wanless et al., 2011; Birgisdóttir et al., 2015; Gross, 2015).

Recently, McClelland et al. (2015) formulated a framework for the study of self-regulation based on the notion of *relational developmental systems* (Overton, 2013), in which development, as a whole, is conceived as a dynamic and bidirectional process of person-context relations that regulate each other: The context provides conditions for regulation and regulation alters the context in a bidirectional relationship. Thus, the concept of self-regulation implies an active individual who affects the context in ways that in turn regulate their behavior, which can occur either intentionally or automatically (McClelland et al., 2015).

Accordingly, the study of self-regulation should be characterized by the acknowledgment that the efforts of an individual to modify internal processes and behaviors to achieve goals are immersed in a cultural context that gives priority to prevailing values and outcomes in the socialization process. In this regard, Trommsdorff (2009) states that the expectations of significant others become an integral part of children’s value systems, which lead them to develop a self-regulation style that aims to fit into the culture to which they belong.

## The Role of Culture for the Development of Children’S Self-Regulation

### Cultural Models

Markus and Kitayama (1991) suggest a theoretical framework for the function of independence and interdependence. While independence is associated with the constitution of a strong sense of self that is autonomous and clearly separated from others, interdependence emphasizes the relational nature of individuals, that is, the establishment of harmonic relationships within the reference group and the effective contribution to the achievement of community goals.

Moreover, cultural values shape socialization theories, goals, and practices (Keller and Kärtner, 2013) which in turn form a developmental niche (Super and Harkness, 1997) that mediates the influence of culture on children’s development. Thus, the values and shared ideas about expected socialization outcomes affect the learning of behaviors that are considered desirable (Harwood et al., 1996; Albert and Trommsdorff, 2014), contribute to the configuration of a sense of self (Markus and Kitayama, 2010), and influence the development of self-regulation (Trommsdorff et al., 2012).

### Transmission of Cultural Models and Self-Regulation

Higgins (1998) and Higgins et al. (2008) proposed the concepts “ideal-self” and “ought-self” which are relevant for a culture-sensitive perspective on the socialization of self-regulation. The “ideal-self” represents the attributes that someone would like to possess. It refers to a desirable or pleasant state which the individual tries to approach through a set of actions. Goals related to the ideal-self generate a promotion-focused self-regulation. Accordingly, attention is centered on accomplishment and success. The “ought-self” implies the representation of attributes that someone believes a person should or ought to possess. Goals related to the ought-self lead to a prevention-focused self-regulation (safety, responsibility, and meeting obligations). Although promotion and the prevention systems exist in each culture, there might be differences among cultures in the strength of their application. For instance, cross-cultural studies have shown that in Asian cultures, prevention-focused self-regulation tends to prevail, whereas in Western cultures, there might be a stronger promotion focus (Higgins et al., 2008; Trommsdorff, 2009).

These findings are in accordance with the notion that independence and interdependence values are important in any culture, but they differ in the sense of being more or less prevailing in distinct aspects of daily life, including the way in which parents socialize their children (Harwood et al., 2001; Leyendecker et al., 2002; Jing-Schmidt, 2014) and promote self-regulation according to the cultural context (Trommsdorff, 2009). In other words, cultural groups might differ in the emphasis that parents put in their educational practices and how they instill their associated values (Neef, 2003). Consequently, self-regulation can satisfy the needs of the individual or the group. In the first case, self-regulation is considered as a motivated action, aimed to achieve autonomy by influencing other people or environmental factors, depending on one’s own needs and goals. When self-regulation is based on group goals, the focus is on flexibility and adjustment of the self to the expectations of other people and social relations, which requires an effective interpersonal regulation (Trommsdorff, 2009).

### Socialization Practices and Self-Regulation in Cultural Contexts

With regard to children’s development of self-regulation, it is noted that self-regulation patterns reflect the history of availability and parenting practices of caregivers as a source of regulation (Calkins and Hill, 2007). In particular a positive parental control has been shown to play an important role for the development of children’s self-regulation (Karreman et al., 2006). Parenting practices are displayed in a cultural context in which it is considered desirable for the individual to self-regulate, especially when personal and relational goals are in conflict (Trommsdorff and Cole, 2011). Moreover, parenting practices affect children’s appraisal of their experiences, the identification of the appropriate timing to regulate their emotions as well as the selection and implementation of specific strategies and behaviors (Díaz and Eisenberg, 2015). In this process, parents shape regulation skills and communicate norms and expectations, guided by intuitive theories about socioemotional competence (Trommsdorff and Cole, 2011).

Thus, the development of the ability and motivation to self-regulate is closely related to the motivation and intention to act in accordance with the expectations of others. These expectations in turn are influenced by cultural values (Trommsdorff, 2009). More specifically, when trying to meet the expectations of their caregivers, children internalize social values and rules that reflect the cultural environment in which the family is embedded (Grusec and Goodnow, 1994). These social values and rules help to understand the intentions and actions of others and to structure the direction and strength of self-regulation (Heikamp et al., 2013). Thus, the kind of control that parents exert is not only relevant for the development of children’s self-regulation in general (Karreman et al., 2006), but also for individual differences in the selection and implementation of regulatory strategies in specific contexts (Díaz and Eisenberg, 2015).

As self-regulation might be understood differently, depending on the parental theories, goals, and practices to which children are exposed during their socialization process, there might be different ways to conceive and foster self-regulation in different cultural groups. Therefore, it may be expected to find different outcomes in terms of children’s behaviors, emotions, and cognitions in different cultural contexts, in situations in which children are supposed to self-regulate. This approach allows the understanding of differences among individuals from different cultural groups as well as the uniqueness of individual development within a given cultural context. In the following paragraphs, studies on the socialization of children’s self-regulation in different cultural contexts are reviewed.

## Studies on the Socialization of Children’S Self-Regulation in Cultural Contexts

### Socialization Goals and Practices in Cultural Contexts

Self-regulation, understood in terms of group goals, is closely connected to attending and fulfilling social expectations, rules, rituals, and roles. So, in interdependent contexts this is perceived as a support for self-regulation and a way to give children a strong sense of belonging to the group. In contrast, in cultures that promote an independent self, external rules and obligations might be experienced as a way of coercive control that undermines autonomy and self-regulation (Trommsdorff, 2009).

Cross-cultural studies have shown that in Asian cultures, the regulation of behavior, emotions, and cognitions is generally subordinated to the preservation of social harmony, while in the European American culture, self-regulation serves to improve the autonomy of the individual and the opportunities to fulfill personal goals (Trommsdorff, 2012). The two positions imply different views of the self as an agent that develops, either in narrow connection and interdependence with others, or as a separate, unique entity. In the first case, the self is malleable and its actions are understood as conjoined with the actions of others to maintain community ethics. Values of duty, respect, and obligation acquire great relevance because they allow to adjust personal goals to the goals and expectations of others. In the second case, the self is considered as individual and fixed, expressing itself and reaching its own goals, so the emphasis is in the differentiation, but not in the coordination among selves.

In the same line, Rothbaum and Wang (2010) have found two different focuses of socialization goals of parents in Western and Eastern countries. In Western countries, children are socialized toward primary control, which means that they see the world as changeable, whereas the individual self is perceived as something fixed. Thus, the world may be transformed, so that it adapts better to the individual. This vision is translated into specific socialization goals such as independence, assertiveness, and self-realization. In contrast, in East Asian countries, children are socialized toward secondary control. That means to conceive of the world as fixed and the individual self as changeable. Thus, the self must change to adapt to the world, and this is related to socialization goals such as obedience, self-control, and cooperation (Rothbaum and Wang, 2010).

Two other studies (Harwood et al., 1999; Carlson and Harwood, 2003) compared socialization goals and practices of European American and Puerto Rican mothers of under 2-year-old children. Both studies found that European American mothers focused their attention mainly on providing their children with opportunities for individual achievement and development of autonomy (i.e., independence), whereas Puerto Rican mothers gave special value to their children becoming decent people, who knew to behave properly in society. In line with their socialization goals, European American mothers preferred educational practices that gave their children learning opportunities. They structured learning situations indirectly, as they used suggestions and verbal approvals to guide children to act in a certain way, while giving them much room to choose for themselves what to do exactly. Puerto Rican mothers, instead, tended to use more authority to teach their children the ways of acting, according to what they expected. They intervened physically more often (e.g., by moving their children to do something), used more explicit signals to obtain children’s attention, and gave more direct orders than European American mothers.

Further, cross-cultural studies showed that, while in the European American context the authoritative parental style helps to promote emotional adjustment of children and adolescents, the promotion of emotional adjustment in the Asian and Latin American contexts is achieved through a more authoritarian style (Chao, 1994; Carlson and Harwood, 2003; Ang and Goh, 2006; Huang and Gove, 2015), and in other contexts (e.g., Spain) it might be achieved through an indulgent style (Fuentes et al., 2015).

### Emotional Self-Regulation in Cultural Contexts

Studies concerning emotional regulation have reported that in Western countries, like Germany, parents usually encourage their children to express emotions such as dissatisfaction or anger because they assume that this will contribute to their development as self-assertive individuals who are capable of recognizing and showing their own needs. In contrast, in Eastern countries, like India, parents prefer to undermine such reactions by downplaying the frustrating event and supporting their children to accept the situation. In this way, children learn to restrict the expression of negative emotions and thus do not perturb interpersonal harmony (Trommsdorff et al., 2009, unpublished).

In the same line, it has been reported that while Asian parents promote a sense of self through which children learn to practice self-reflection and self-criticism as a mean to change themselves and to adequately fulfill their duties toward their family and reference group, European parents and European American parents want their children to find their own way of being, to find their unique self, and to express themselves in front of others (Trommsdorff, 2012).

Heikamp et al. (2013) examined in a study in Germany, India, Nepal, South Korea, and the United States, maternal responses to children’s negative emotions. While European American and German mothers encouraged children’s emotional expressivity, Indian and Nepalese mothers experienced distress from their children’s negative emotions and “preferred to intervene proactively in order to avoid children becoming upset” (Heikamp et al., 2013, p. 211). The authors conclude that in independence-oriented socialization contexts, parents intentionally foster the expression of “socially disengaging” emotions because this is in line with the shared cultural values of individuality, authenticity, and autonomy. In contrast, parents from cultural contexts that highly value interdependence and relatedness, promote the expression of “socially engaging” emotions (e.g., positive emotions toward others) because they help to preserve the social harmony.

The different emphases on the kind of socialization practices for promoting emotional self-regulation in children could help to explain differences in the suppression of emotional expressions between Asian and European American adults, as documented in a recent study by Murata et al. (2013). The authors argue “…that because Asians do not value expression of the inner self in general and that of emotional experience in particular, they are likely to learn, through ‘cultural training’, to attenuate emotional processing when they are required to suppress their emotional expression” (Murata et al., 2013, p. 598).

### Behavioral Self-Regulation in Cultural Contexts

Some studies have reported that developmental processes, which are related to behavioral self-regulation, can take place earlier or later in children’s development depending on the prevalence of specific socialization goals in a cultural context. For instance, Keller et al. (2004) assessed the development of self-recognition and behavioral self-regulation in 18- to 20-month-old children from Nso rural families in Cameroun and middle class Greek and Costa Rican families. The three samples represented different sociocultural models. Nso families had a low socioeconomic and educational profile and were strongly oriented to interdependence. Children were expected to become communal agents, interconnected with others, role-oriented, and compliant. They should fulfill responsibilities in the household and care for younger children from an early age. Greek families, in turn, had a high educational level and were mainly oriented toward independence. They promoted the development of children as individual agents, self-contained, unique, and separate from others. Costa Rican families, also characterized by high educational levels, valued both independence and interdependence highly. Costa Rican children were expected to become financially independent but to remain very close to their family.

In Keller et al.’s study, self-regulation was understood as following instructions given by an adult. It was shown that Nso children followed the instructions at the youngest age, followed by Costa Rican children. This finding makes sense considering that obedience and cooperation with adults are essential to respond adequately to the expected roles of children in the family or reference group. On the other hand, self-recognition, assessed through the identification of one’s own face in a mirror, was developed earlier in Greek children, followed by Costa Rican children. This is consistent with the greater emphasis placed by parents in the development of independence.

Other cross-cultural studies have used children’s compliance as an indicator of behavioral self-regulation, especially in early childhood. For instance, Feldman et al. (2006) examined 5- to 33-month-old Palestinian and Israeli children in “do” and “don’t” compliance tasks. Although both groups of children showed similar levels of self-regulation, the Israeli children performed better in “do” and Palestinian children in “don’t” compliance tasks. Values and parenting practices differed in both groups. Israeli parents emphasized “self-expression,” “creativity,” “assertiveness,” and “intelligence” of their children as socialization goals and preferred social gazing, active touch, and indirect assistance (suggestions) to encourage obedience. In contrast, Palestinian parents valued “compliance,” “respect for elders,” “quiet,” and “polite” to a higher degree and used prolonged physical contact in infancy or direct assistance in toddlerhood to foster their children’s obedience.

## Studies on Specific Aspects of Self-Regulation in Cultural Contexts

### Executive Functions in Cultural Contexts

Most studies on executive functions do not report any systematic references or an emphasis on cultural variations in executive functions. However, during the last decade, a growing number of researchers have begun to address questions about the context-sensitive nature of self-regulatory processes involved in executive functions. For instance, Moriguchi et al. (2012) reported that executive functions can be influenced by another individual’s executive actions and that these social influences might vary in different cultural contexts. They found that 3- and 4-year olds in Japan and Canada performed similarly in an executive functioning task when they were not exposed to a model’s behavior. However, when a model was introduced during the task, Japanese children were more strongly influenced by the model’s behavior than Canadian children. The authors attributed this finding to the fact that in Japan children are socialized from early infancy to be interdependent with other members of their social environment, so their executive actions may become more attuned to similar actions of others, compared to Canadian children, who are socialized in a more independent society (Moriguchi et al., 2012).

In a similar vein, Imada et al. (2013) examined 4- to 9- year old children in the United States and Japan and found that context-sensitivity in a set-shifting executive function task increased with age across cultures, especially in Japanese children. According to these researchers, North Americans tend to focus on a central object in a visual scene, whereas East Asians are more attentive to the context, probably because in the North American culture, sensitivity to situational cues is not as crucial as it is in East Asian cultures such as Japan, where people are expected to adjust themselves to social situations to preserve group harmony. Thus, these cultural differences in context-sensitivity might reflect and account for specific cultural differences in self-regulation.

Other researchers have also shown that executive functions are related to social experiences and cultural factors. For example, Lewis et al. (2009) conducted studies in Korea, China, and Japan, which suggest that social and executive skills might be influenced by cultural processes as different societies embrace distinct parenting practices that are crucial to the development of human skills. Particularly, they reported that the patterns of executive skills and false belief measures in Korea, China, and Japan differ from those observed in Westerners. A possible explanatory factor for the development of cultural differences in executive skills might be the variety of parental demands and practices in terms of the control of behavior in distinct cultural contexts.

Preschoolers from Asia have been found to perform better on executive-functioning tasks than their Western counterparts (e.g., Sabbagh et al., 2006; Lewis et al., 2009). Based on these results, Ellefson et al. (2017) examined whether this advantage persists beyond childhood. They asked middle school students from Hong Kong and the United Kingdom and their parents to complete four executive-functioning tasks assessing inhibition, shifting, planning, and working memory. The study revealed that children - but not parents - in Hong Kong outperformed their peers in the United Kingdom. Executive-functioning efficiency increased with age at a similar rate for children from both locations. In addition, the researchers found a small but significant correlation in executive-functioning skills between children and their parents. These findings suggest that the differences in executive-functioning observed in early childhood are still present in early adolescence; they could reflect differences in the socialization practices of parents from the different cultural groups.

### Development of Attention in Cultural Contexts

Attentional processes are important for self-regulation as they allow the individual to handle emotional reactivity by amplifying or modulating levels of arousal, supporting the intentional and volitional control of self-directed behavior, limiting impulsive responses, regulating emotions, solving problems, and planning ahead (Blair, 2016). Some researchers have documented cultural differences in attentional processes via contextual information. For instance, Kuwabara et al. (2011) showed that children from the United States interpreted facial expressions without considering the context in which they appeared, whereas Japanese children were more prone to shift their judgments according to changes in the context. Based on these results, Kuwabara et al. (2011) point to the need of studies about the influence of parental practices in independent vs. interdependent cultures on the way children use attentional processes in emotional tasks.

Related research has been reported by Chavajay and Rogoff (1999), who compared mother-child dyads from Mayan and European American cultures and observed different patterns in the direction of attention. Mayan mothers and children attended more often to competing events simultaneously, while European American mothers and children alternated their attention more often. Similarly, Correa-Chávez et al. (2005) observed 6- to 10-year-old children in classroom settings and found that children of Mexican mothers with indigenous education were more likely to attend simultaneously to several events, while children of European American mothers with high levels of formal education, were more likely to apply their attention to one event at a time. In addition, Silva et al. (2010) found that children of Mexican mothers with indigenous education paid more attention to their peers while performing a task and then needed less help to resolve it, compared to children of Mexican mothers with advanced levels of formal education.

Senzaki et al. (2016) indicate that mothers and other experienced adult members of a given culture play an essential role in the communication of culturally dominant modes of attention. However, according to these authors, little is known about how differences in attentional processes are driven by culture and socialization processes. In their studies, Senzaki et al. (2016) found that Canadian and Japanese children in the age of 4–9 years did not differ in a scene description task while working independently. However, by age 9, a culturally unique mode of attentional pattern appeared to emerge, when performing the same scene description task in the presence of parents. Canadian parents referred more often to focal objects than did Japanese parents, and Japanese parents referred more often to the background than did Canadian parents. Moreover, parents communicated to their children differently across cultures. The descriptions performed by the older group (7–9 years old) showed significant cross-cultural differences in attention (object-oriented mode of attention in Canada and context-sensitive mode of attention in Japan), while the focus of attention among the younger group (4–6 years old) did not differ.

In sum, according to these findings, parents transmit their culturally unique mode of attention to their children, providing them with opportunities for cultural learning and skill acquisition (Senzaki et al., 2016).

### Emotional Display Rules in Cultural Contexts

A specific aspect of emotion regulation, in which cross-cultural factors have been studied already, is the study of emotional display rules. Emotional display rules are defined as cultural prescriptions, which influence the emotional experience, guiding the individual in what is considered as acceptable in terms of emotional expressions that might differ from the underlying emotional state (Safdar et al., 2009). This dimension of emotional self-regulation is susceptible to cultural variations. Matsumoto et al. (2008) conducted a study with more than 5,000 participants in 32 countries. They found an overall regulation effect among individuals in all countries. However, individualistic and collectivistic countries differed in the norms about specific emotions in in-group and out-group situations. In the same line, Novin et al. (2009) found that compared to a Dutch sample, Iranian children tended to mask emotions in front of family members, and they mentioned both prosocial and self-protective motives for concealing emotions. However, during interactions with peers, Iranian children concealed emotions less often than Dutch children.

## Conclusions and Future Directions

Despite the reported pioneering works on self-regulation in cultural contexts, evidence about cross-cultural differences in specific mechanisms of emotional and behavioral regulation is still limited. There seems to be at least two reasons why the context-sensitive nature or cultural specificity of self-regulation has not been thoroughly addressed.

First, self-regulation has been approached mainly from a biological and maturational perspective (Blair and Raver, 2015), without enough attention paid to cultural variation in developmental paths. When this consideration appears, it seems to be a loose enunciation of the “cultural factor” without appropriate specificity in the explanations. Furthermore, the evidence usually comes from adult samples, without enough attention to cultural specific tendencies developed through early socialization or the developmental course of context-sensitivity (Imada et al., 2013). Second, the field needs to make more room for a conception according to which human beings have evolved to take adaptive advantage from living in large groups. To accomplish this evolutionary goal, humans have had to develop a series of skills such as self-regulation, which may vary depending on the characteristics of the social environment, which in turn is transformed as a function of the self-regulatory characteristics of the individuals (Lindenberg, 2015).

In sum, the interest on the cultural specificity of self-regulatory processes has only recently begun to grow and consolidate.

The study of self-regulation has raised great interest because of its importance for children’s education achievement and their preparation to achieve goals and to participate successfully in different contexts of social life, such as school, peer group, and home. Moreover, self-regulation might contribute to a feeling of satisfaction, harmony, and control over one’s own existence and well-being.

As noted above, self-regulation might be understood and socialized differently in distinct cultural contexts. However, more research is needed on the intra-cultural and inter-cultural variability of the relations between parental socialization goals, practices, and children’s self-regulation. Cross-cultural research can contribute to a better understanding of the interaction between environmental demands and personal resources in the development of self-regulatory processes (McClelland et al., 2015).

So far, research has been successful in describing cultural differences in socialization theories, goals, and practices as well as in describing the development of children’s self-regulation. However, more research is required about the specific mechanisms through which these parental theories, goals, and practices, and children’s self-regulatory processes influence each other.

A contextual and culture-sensitive approach demands the investigation of regulatory processes in interpersonal interactions that prepare the development of self-regulation during early childhood. Some researchers have introduced the notion of co-regulation to refer to mutual regulation processes, which take place in dyads when exposed to disturbances or conflicts that force individuals to seek ways to self-regulate and achieve a common goal (Lunkenheimer et al., 2016). These co-regulatory processes seem to be particularly relevant in preschool years, when self-regulatory capacities of children are still developing. During this developmental phase, parents often cooperate to help children to achieve a goal that requires self-regulation, by sharing intentions, emotions, and strategies that contribute to improve their self-regulatory skills in a way that is consistent with accepted values in the cultural context. This kind of co-regulation includes two or more individuals (e.g., mother and child), who cooperate to achieve a common goal. However, the interaction is asymmetrical as it is directed by the adult, in accordance to parental goals and theories. The child uses strategies provided by the adult and progressively applies them through sustained interaction. It is supposed that this process occurs in any socialization context. However, more research is needed about the particularities of co-regulation interactions in distinct cultural groups.

In conclusion, this contribution points to the importance of contextual and culture-sensitive approaches on the development of self-regulation and aims to encourage researchers to develop methodological designs focusing on reciprocal relations between culture, parenting, and children’s self-regulation.

## Author Contributions

JJ and MR are the principal authors of this article. They contributed to the conception of the article and to the search, review, and critical analysis of scientific literature on the subject. They wrote a first version of the manuscript and introduced new sections and changes proposed by the others authors. Finally, they realized a final version of the manuscript and adjusted it several times taking into account the suggestions of the other authors. LM and MW contributed to the conception, to the search, review and critical analysis of scientific literature on the subject. They reviewed the different versions of the manuscript, suggesting some changes each time and writing several additional paragraphs. They approved the final version of the document. GT shared with the other authors her long experience of research on the development of self-regulation in children from a cultural perspective. She offered guidelines for the conception of the article and the critical revision of the scientific literature. Likewise, she proposed changes in several sections of the manuscript and helped to reformulate it.

## Conflict of Interest Statement

The authors declare that the research was conducted in the absence of any commercial or financial relationships that could be construed as a potential conflict of interest. The reviewer AMM and handling Editor declared their shared affiliation, and the handling Editor states that the process nevertheless met the standards of a fair and objective review.
